# Poly(glycerol monomethacrylate)-encapsulated upconverting nanoparticles prepared by miniemulsion polymerization: morphology, chemical stability, antifouling properties and toxicity evaluation†

**DOI:** 10.1039/d3na00793f

**Published:** 2023-11-13

**Authors:** Taras Vasylyshyn, Vitalii Patsula, Marcela Filipová, Rafal Lukasz Konefal, Daniel Horák

**Affiliations:** a Institute of Macromolecular Chemistry, Czech Academy of Sciences Heyrovského nám. 2 162 00 Prague 6 Czech Republic horak@imc.cas.cz

## Abstract

In this report, upconverting NaYF_4_:Yb^3+^,Er^3+^ nanoparticles (UCNPs) were synthesized by high-temperature coprecipitation of lanthanide chlorides and encapsulated in poly(glycerol monomethacrylate) (PGMMA). The UCNP surface was first treated with hydrophobic penta(propylene glycol) methacrylate phosphate (SIPO) to improve colloidal stability and enable encapsulation by reversible addition–fragmentation chain transfer miniemulsion polymerization (RAFT) of glycidyl methacrylate (GMA) in water, followed by its hydrolysis. The resulting UCNP-containing PGMMA particles (UCNP@PGMMA), hundreds of nanometers in diameter, were thoroughly characterized by transmission (TEM) and scanning electron microscopy (SEM), dynamic light scattering (DLS), infrared (FTIR) and fluorescence emission spectroscopy, and thermogravimetric analysis (TGA) in terms of particle morphology, size, polydispersity, luminescence, and composition. The morphology, typically raspberry-like, depended on the GMA/UCNP weight ratio. Coating of the UCNPs with hydrophilic PGMMA provided the UCNPs with antifouling properties while enhancing chemical stability and reducing the cytotoxicity of neat UCNPs to a non-toxic level. In addition, it will allow the binding of molecules such as photosensitizers, thus expanding the possibilities for use in various biomedical applications.

## Abstract 2

Highly colloidally stable upconverting nanoparticles encapsulated in poly(glycerol monomethacrylate) by RAFT miniemulsion polymerization were non-toxic and exhibited antifouling properties.

## Graphical abstract

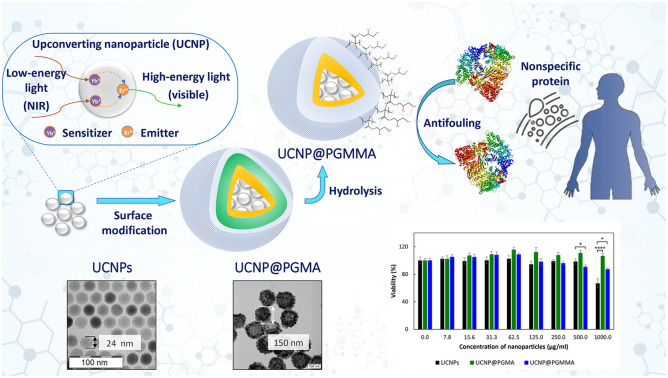

## Introduction

Light-emitting nanomaterials have long been investigated in various fields of biomedicine.^1^ As an example, luminescent probes, typically organic dyes and fluorescent peptides, have made it possible to localize proteins, monitor biological processes, and detect cancer biomarkers, cells, tissues, *etc.*^2^ Fluorescent organic dyes have several advantages over other luminescent materials due to their small size, biocompatibility, relatively high fluorescence intensity, and easy modifiability for covalent conjugation of biomolecules.^3^ However, their use is limited by the short detection time due to photobleaching and chemical degradation. In addition, organic dyes often have narrow absorption bands and broad emission spectra with tailing, which again limits their detection.^4^

Lanthanide-doped upconverting nanoparticles represent a new class of fluorophores with the unique ability to overcome the above disadvantages. These particles are based on three main components: a host matrix, a sensitizer and an emitter (activator). Upon excitation, the sensitizer absorbs the incoming photon in the near-infrared region and transfers the energy to the nearby emitter, resulting in upconversion emission. Each trivalent lanthanide activator/emitter pair has an individual energy level structure and produces emission peaks at specific wavelengths, which depends on the ion concentration and dopant/activator ratio.^5–7^ Typically, upconverting nanoparticles have different sizes (10–200 nm) and morphologies (triangular, quadrangular, hexagonal, spherical, star- or rod-shaped, *etc.*) based on the synthesis method.^8^ These include thermal decomposition, coprecipitation, hydrothermal or microemulsion template synthesis, sol–gel, and ionic liquid-based methods.^9^ The excellent properties of upconverting nanoparticles, such as variable excitation dynamics, large anti-Stokes shift, sharp emission bands, low autofluorescence, deep tissue penetration of near-infrared (NIR) light, and low photodamage to surrounding biological tissues, can be easily tuned to meet the requirements of a specific aim.^10,11^ In addition, lanthanide-doped upconverting nanoparticles provide a long luminescent lifetime and good photochemical stability.^12^

The combination of all the above advantages makes upconverting nanoparticles a promising tool for various bioapplications in precision nanomedicine, *e.g.*, for deep tissue *in vivo* imaging, photoacoustic molecular imaging, drug and gene delivery, background-free biosensing, visual neurophysiology, optogenetics or photothermal and photodynamic therapy.^13–16^ On the other side, there are also some drawbacks associated with these materials that hinder their clinical use, such as particle aggregation, unknown interactions with biomolecules, disintegration in buffers and biological fluids, and so-called “dark” toxicity due to the subsequent release of toxic lanthanide and fluoride ions.^5,12,17^ Another important factor affecting the *in vivo* performance of upconverting nanoparticles in diagnostics and therapy is biofouling, which must be suppressed if enhanced targeting and drug delivery is to be achieved.^18^

To avoid the aforementioned adverse effects and prevent biofouling, various strategies of particle surface modification have been proposed.^18–20^ An interesting approach is the formation of core–shell compositions *via* miniemulsion polymerization of glycidyl methacrylate (GMA) in the presence of dispersed upconverting nanoparticles. The uniqueness of this robust technique lies in the nucleation inside monomer droplets, as opposed to micellar or homogeneous nucleation that dominates macroemulsion polymerization.^21^ Moreover, the miniemulsion droplets are prepared in the presence of surfactant and hydrophobe (typically hexadecane) to suppress Ostwald ripening. The resulting particle morphology then depends on both kinetic and thermodynamic aspects of the polymerization, including polymerization time and temperature, molar mass of reagents and monomer concentration.^22,23^ For example, hollow structures can be formed by differences in interfacial tension and phase separation during polymerization due to the immiscibility of hydrophobe and polymer. Such structures are useful for opaque pigments, glossy paper coatings, high-resolution inks, or pharmaceuticals.^22^ The advantage of GMA used in this report lies in the presence of reactive oxirane groups that can undergo ring-opening reactions with biomolecules or hydrolysis to hydrophilic poly(glycerol monomethacrylate) (PGMMA). PGMMA is a highly biocompatible water-soluble polymer that is non-fouling and has been previously used as a biomaterial for soft contact lenses or as a stealth agent for the delivery of therapeutic compounds as an alternative to PEG.^24–26^ PGMMA has very low toxicity, limited protein interactions, minimal immunogenicity and can be easily functionalized to bioconjugate a variety of compounds. In contrast, poly(glycidyl methacrylate) (PGMA) is widely used for the industrial production of epoxy functional methacrylic resins, coatings and adhesives. In order to control the molecular weight and polydispersity of PGMA in composite particles, reversible addition–fragmentation chain transfer (RAFT), a kind of living radical polymerization, was chosen. It can be easily implemented in different reaction modes (*e.g.*, miniemulsion) and chain transfer agents (CTAs) used are relatively cheap. They can also be designed as macromolecular CTAs (macroCTAs), which can be bi- or multifunctional,^27^ enabling various innovative approaches, such as photo-induced electron/energy transfer-RAFT (PET-RAFT), electro-RAFT, sono-RAFT, and photo-RAFT.^28–31^ The aim of this report was to introduce a PGMMA shell around upconverting nanoparticles that could prevent the leakage of Ln^3+^ and F^−^ ions into biological fluids and potentially be further modified, *e.g.* by photosensitizers. The effect of the reaction parameters on the particle morphology was then investigated, enabling prospective use of these composite particles as a multimodal luminescent material with low toxicity and biofouling and high chemical and dispersion stability in biological media.

## Experimental

### Materials

Octadec-1-ene (90%), ammonium fluoride (99.99%), anhydrous yttrium(iii) and ytterbium(iii) chlorides (99.9%), erbium(iii) chloride hexahydrate (99.99%), 4-cyano-4-(phenylcarbonothioylthio)pentanoic acid (CPCTPA; chain transfer agent CTA; 98%), l-ascorbic acid sodium salt (NaAs; 99%), 4,4′-azobis(4-cyanovaleric acid) (ACVA; ≥75%), potassium persulfate (KPS), phosphate buffered saline (PBS; pH 7.4), and Dulbecco's Modified Eagle's Medium (DMEM) were purchased from Sigma-Aldrich (St Louis, MO, USA). KPS and ACVA were recrystallized before use. Artificial lysosomal fluid (ALF; pH 4.5) was prepared according to the literature.^32^ Oleic acid (OA), methanol, toluene, tetrahydrofuran (THF), chloroform, hexane, and dichloromethane (DCM) were obtained from Lach-Ner (Neratovice, Czech Republic). Glycidyl methacrylate (GMA) and fluorescein isothiocyanate (FITC) were purchased from Fluka (Buchs, Switzerland); GMA was vacuum-distilled before use. Penta(propylene glycol) methacrylate phosphate (Sipomer PAM 200; SIPO) was obtained from Rhodia (Courbevoie, France). 3-(4,5-Dimethylthiazol-2-yl)-2,5-diphenyl-2*H*-tetrazolium bromide (MTT) assay was purchased from Abcam (Cambridge, UK). Bovine serum albumin fraction V (BSA; *M*_w_ = 67 kg mol^−1^) was purchased from Serva Electrophoresis (Heidelberg, Germany). BSA-FITC was prepared according to an earlier publication.^33^ Other chemicals were purchased from Sigma-Aldrich and used as received.

### Synthesis of NaYF_4_:Yb^3+^, Er^3+^ upconverting nanoparticles (UCNPs)

NaYF_4_ nanoparticles doped with Yb^3+^ (20 mol%) and Er^3+^ (2 mol%) ions were synthesized by high-temperature OA-stabilized coprecipitation of lanthanide chlorides in octadec-1-ene as a high-boiling organic solvent.^34^ Briefly, YCl_3_ (0.78 mmol), YbCl_3_ (0.2 mmol), ErCl_3_·6H_2_O (0.02 mmol) were added to a 100 ml three-necked round-bottom flask, which was subsequently loaded with OA (12 ml) and octadec-1-ene (30 ml). The mixture was heated at 160 °C for 30 min with stirring (250 rpm) in an Ar atmosphere to form a transparent yellowish solution, which was cooled to room temperature (RT). A methanol solution (10 ml) of NaOH (4 mmol) and NH_4_F (2.5 mmol) was added dropwise to the flask, and the mixture was slowly heated at 80–90 °C until the methanol evaporated and then at 300 °C for 1.5 h. After cooling to RT, the UCNPs were precipitated in ethanol and separated by centrifugation (3460 rcf) for 30 min. After washing three times with ethanol, the UCNPs were dispersed in 10 ml of hexane.

### Synthesis of RAFT agent

#### Preparation of glycerol monomethacrylate (GMMA)

GMMA was synthesized according to the reported procedure.^35^ A solution of GMA (0.08 mol) in THF (40 ml) and 0.5 M H_2_SO_4_ (100 ml) was added to a 250 ml round-bottom flask and the mixture was stirred at RT for 4 h. After cooling the reaction mixture to 5 °C for 16 h, GMMA was three times extracted with DCM (100 ml) in a separation funnel, the organic layers were combined, dried with MgSO_4_, and the DCM was removed in a rotary evaporator at 25 °C and 133 Pa.

#### Preparation of PGMMA-macroRAFT agent (Fig. 1a)

Briefly, CPCTPA (0.92 mmol), GMMA (45.6 mmol), ACVA (0.184 mmol; CTA/ACVA = 5 mol mol^−1^), and ethanol (11.3 ml) were added to a 25 ml round-bottomed flask, which was sealed and purged with Ar for 30 min. The flask was immersed in an oil bath at 70 °C with stirring (700 rpm). After 100 min of reaction, GMMA polymerization was stopped by exposing the mixture to air, cooled for 2 min in dry ice and diluted with methanol (20 ml).

**Fig. 1:**
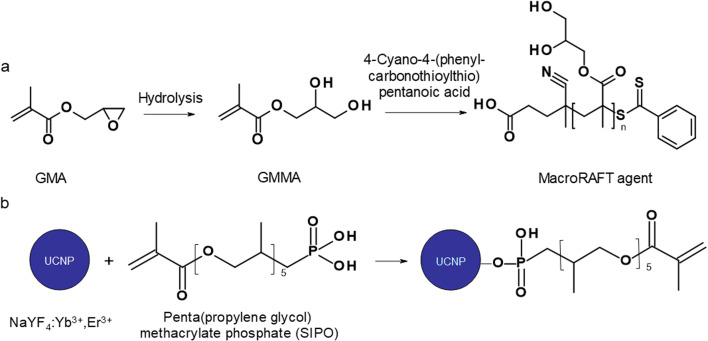
(a) Preparation of poly(glycerol methacrylate)-based macroraft agent and (b) surface modification of UCNPs with SIPO.

### Surface modification of UCNPs

#### Coating of UCNPs with SIPO (Fig. 1b)

UCNPs were first washed with hexane/ethanol mixture (5/1, 2/1, 1/1, 1/2, and 1/5 v/v), ethanol, ethanol/water mixture (5/1, 2/1, 1/1, 1/2, and 1/5 v/v) and water to remove OA and redispersed in water by sonication (Bandelin Sonoplus; Berlin, Germany; 15% power) for 5 min. The aqueous dispersion of UCNPs (100 mg particles; 12 ml) was added to a solution of SIPO (160 mg) in toluene/chloroform (1/1 v/v; 8 ml) under stirring (1500 rpm) in an Ar atmosphere and the reaction was run at RT for 16 h. Then, the organic layer was separated and the solvents were removed at 30 °C and 133 Pa using a rotary evaporator. To wash the SIPO residues from the UCNPs, these were redispersed in chloroform, centrifuged (3461 rcf) for 30 min and the UCNP@SIPO particles were transferred to DCM.

#### Reversible addition–fragmentation transfer (RAFT) miniemulsion polymerization

Briefly, GMA (100 mg), dispersion of UCNP@SIPO in DCM (1 ml) containing additional SIPO (UCNP/SIPO = 3 w/w), macroRAFT CTA (6.35 μmol) and water (0.72 ml) were loaded into a 2 ml round-bottom flask, the mixture was cooled in an ice bath for 5 min under sonication (50% power), DCM was removed at 25 °C using a rotary evaporator, the system was degassed with Ar at 25 °C for 20 min, and aqueous solutions of redox initiators KPS (70 μl; 4 mg ml^−1^) and NaAs (69 μl; 3 mg ml^−1^; NaAs/KPS/CTA 1 : 1 : 5 mol mol^−1^ mol^−1^) were injected with a Hamilton syringe. The mixture was magnetically stirred at 50 °C for 4 h until full monomer conversion was achieved and polymerization was quenched by exposure to air and the addition of hydroquinone (10 mg). The resulting particles were denoted as UCNP@PGMA.

#### Hydrolysis of UCNP@PGMA particles to UCNP@PGMMA particles

1-Mehylpyrrolidone (1 ml), 1,4-dioxane (1 ml) and 96 wt% sulfuric acid (0.14 ml) were added to an aqueous dispersion of UCNP@PGMA particles (5 ml; 10 mg ml^−1^) and the mixture was stirred at RT for 48 h. Resulting UCNP@PGMMA particles were separated by centrifugation (3460 rcf) for 30 min, washed three times with water and freeze-dried.

### Characterization of particles

The composition and purity of GMA and macroRAFT CTA were analyzed by ^1^H and ^13^P NMR spectroscopy on a Bruker Avance III 600 spectrometer (Bruker; Billerica, MA, USA). The monomer conversion was determined from the polymerization mixture in D_2_O by ^1^H NMR spectroscopy. Before measurement, the UCNP particles were removed by centrifugation (13 171 rcf) for 40 min. The morphology of the starting and polymer-encapsulated UCNPs was analyzed using a Tecnai Spirit G2 transmission electron microscope (TEM; FEI; Brno, Czech Republic) and MAIA3 scanning electron microscope (SEM; Tescan; Brno, Czech Republic). The weight-average diameter (*D*_w_ = ΣN_i_·*D*_i_^4^/ΣN_i_·*D*_i_^3^), number-average diameter (*D*_n_ = ΣN_i_·*D*_i_/ΣN_i_) and dispersity (*Đ* = *D*_w_/*D*_n_) were calculated by measuring at least 300 particles from TEM micrographs using Atlas software (Tescan Digital Microscopy Imaging; Brno, Czech Republic). Hydrodynamic diameter (*D*_h_), polydispersity (PD) and *ζ*-potential of particles were determined at 25 °C by dynamic light scattering (DLS) using a ZSU 5700 Zetasizer Ultra (Malvern Instruments; Malvern, UK). The number-average (*M*_n_), weight-average (*M*_w_) molecular mass and dispersity index (*M*_w_/*M*_n_) of the polymers were determined by size exclusion chromatography (SEC) on a Shimadzu HPLC system (Tokyo, Japan) equipped with a UV-vis diode array and OptilabrEX refractive index and DAWN EOS multiangle light scattering detectors (Wyatt; Santa Barbara, CA, USA). Infrared spectra were recorded on a 100T FTIR spectrometer (PerkinElmer; Waltham, MA, USA) using a Specac MKII Golden Gate single attenuated total reflection (ATR). Thermogravimetric analysis (TGA) was performed in oxygen from 30 to 850 °C at a heating rate of 10 °C min^−1^ using a PerkinElmer TGA 7 analyzer (Norwalk, CT, USA). Upconversion luminescence spectra of neat and polymer-coated nanoparticles in water (4 mg ml^−1^) were measured using an FS5 spectrofluorometer (Edinburgh Instruments; Edinburgh, UK) coupled with CW 980 and 808 nm infrared diode lasers as excitation sources with a nominal laser power of 2 W (MDL-III-980; beam size of 5 × 8 mm^2^).

### Chemical stability of UCNP@PGMMA particles

The dispersion of polymer-coated UCNPs (1 mg of neat particles per ml) in 0.01 M PBS (pH 7.4), water, DMEM with 10% fetal bovine serum, and ALF was loaded in 2 ml-plastic vials, closed with rubber seals, and aged at 37 °C for selected time with shaking (250 rpm). The particles were separated by centrifugation (14 129 rcf) for 25 min, resulting supernatants were filtered (MWCO 30 kg mol^−1^) to remove the remaining particles, and the amount of released F^−^ ions was determined as the molar percentage of F^−^ (*X*_F_) relative to the amount of fluorine in the NaY_0.78_F_4_:Yb_0.20_,Er_0.02_ particles using a combined fluoride electrode (Thermo Fisher Scientific; Waltham, MA, USA) according to the manufacturer's protocol.

### Determination of antifouling properties

BSA-FITC solution (0.5 mg ml^−1^) was added to a dispersion of UCNP@PGMA or UCNP@PGMMA particles (1 mg ml^−1^) in PBS (pH 7.4) in a 2 ml Eppendorf flask and the mixture was shaken (250 rpm) at RT for 48 h. Hydrodynamic particle diameter *D*_h_ and polydispersity PD were determined after 0, 1, 6, 24 and 48 h by DLS. The amount of absorbed BSA-FITC on the particles after 0, 1, 3 and 5 days of shaking at RT was determined by an Evolution™ 220 UV-vis spectrophotometer (Thermo Fisher Scientific; Waltham, MA USA). The particles were separated by centrifugation (13 148 rcf) and washed twice with PBS (1 ml); data were analyzed by the OriginLab software.

### Cell viability assay

Human dermal fibroblasts (HFs), provided by the Institute of Experimental Medicine, Czech Academy of Sciences, Prague, were cultivated in DMEM supplemented with 10% fetal bovine serum and 1% penicillin-streptomycin. Cells were seeded in a 96-well plate at a concentration of 8 × 10^3^ cells per well in complete growth medium. After 24 h, neat UCNPs, UCNP@PGMA and UCNP@PGMMA particles (0–1000 μg ml^−1^) were added, allowed to incubate for 24 h and the medium was replaced with MTT (500 μg ml^−1^) dissolved in complete growth medium. After three hours, the medium was removed, the purple formazan crystals were dissolved in dimethyl sulfoxide and the absorbance was measured using a Spark multimode microplate reader (Tecan; Männedorf, Switzerland) at 570 nm. The percentage of viable cells relative to control (100%) was expressed as mean ± standard error of the mean (S.E.M.). Each experiment was performed in triplicate (*n* = 3). Statistical differences between the results were evaluated by one-way analysis of variance (ANOVA) with Dunnett's post-hoc test using Prism version 8.0.1 (GraphPad Software; La Jolla, CA, USA). Statistically significant results were obtained at *p* values <0.05 and <0.0001.

## Results and discussion

### UCNPs

**Fig. 2:**
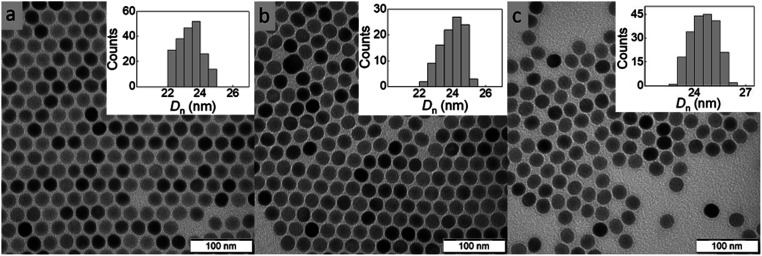
TEM micrographs of (a) OA-stabilized UCNPs in hexane, (b) washed UCNPs in water and (c) UCNP@SIPO in DCM. OA – oleic acid, SIPO – penta(propylene glycol) methacrylate phosphate.

**Fig. 3:**
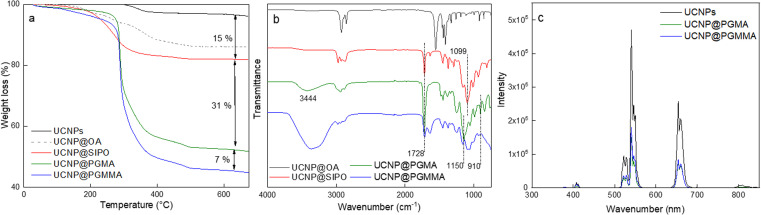
(a) TGA thermograms, (b) FTIR spectra and (c) fluorescence emission spectra of UCNPs, UCNP@OA, UCNP@SIPO, UCNP@PGMMA, and UCNP@PGMA particles. UCNP/SIPO = 3 w/w; UCNP/GMA = 0.5 w/w.

Among the various approaches for the controlled preparation of NaYF_4_:Yb^3+^,Er^3+^ upconverting nanoparticles (UCNPs), the coprecipitation method is preferable due to its simplicity, short reaction time and low cost of reagents. Hence, UCNPs were synthesized by high-temperature (300 °C) coprecipitation of lanthanide chlorides in a high-boiling solvent (octadec-1-ene) in the presence of the OA stabilizer. The morphology of UCNPs examined by TEM microscopy revealed their spherical shape with *D*_n_ = 24 nm and a very narrow size distribution (*Đ* = 1.01; Fig. 2a). The presence of OA (11 wt% according to TGA; Fig. 3a) was also confirmed by FTIR analysis, which revealed peaks at ∼1450 and ∼2900 cm^−1^ belonging to CH and peak at ∼1550 cm^−1^ attributed to COO^−^ groups (Fig. 3b). OA made the particle surface hydrophobic, *i.e.*, dispersible only in organic solvents, and without reactive groups, which would be available for subsequent conjugation with other compounds. In order to make the particles non-aggregating and dispersible in aqueous media, which is one of the main requirements for use in biological systems, they were washed successively with a mixture of hexane/ethanol, ethanol and ethanol/water to remove excess OA while creating conditions for introduction of functional groups that will be available for further reactions. Note that almost no changes in the particle size (*D*_n_ = 24 nm) and dispersity (*Đ* = 1.01) were observed after washing (Fig. 2b). DLS analysis of the washed particles showed that their hydrodynamic diameter (*D*_h_ ∼105 nm; z-average size) was much larger than the number-average (*D*_n_) from TEM due to the effect of charge and the difference between the size of solvated (by DLS) and dry UCNPs (by TEM); moreover, a slight particle aggregation in water cannot be excluded (Table 1). The *ζ*-potential of UCNPs reached 35 mV, while the polydispersity (PD = 0.15) documented a narrow size distribution in agreement with the TEM analysis.

**Table 1:** TEM and DLS analysis of particles^a^^,^^d^

	*D*_n_ (nm)	*Đ*	*D*_h_ (nm)	PD	*ζ*-potential (mV)
UCNPs	24	1.01	105^b^	0.15	35
UCNP@SIPO	25	1.01	60^c^	0.18	—
UCNP@PGMA	150	1.12	259^b^	0.28	−18
UCNP@PGMMA	161	1.13	263^b^	0.29	−22

a *D*_n_ – number-average diameter (TEM), *Đ* – dispersity (TEM).

b *D*_h_ – hydrodynamic diameter (DLS) in water.

c Chloroform.

d PD – polydispersity (DLS). UCNP/SIPO = 3 w/w; UCNP/GMA = 0.5 w/w.

### Surface modification of UCNPs with SIPO

The modification of nanoparticle surface is an important step to prevent ion leakage, make the particles biocompatible, improve their dispersibility and introduce functional groups. In this report, a hydrophobic penta(propylene glycol) methacrylate phosphate (SIPO) protective coating was first attached to the UCNP surface by phosphate bonding (Fig. 1b). SIPO also has the advantage of containing a methacrylate group with a double bond that allows subsequent copolymerization with GMA. Compared to UCNP@OA, SIPO coating had no significant effect on particle size and dispersity (Fig. 2c). According to DLS, the optimal UCNP/SIPO ratio in the synthesis was 1 : 1.6 w/w, where the particle distribution was unimodal, in contrast to the bimodal distribution obtained at 1 : 1 and 1 : 2 w/w ratios (ESI; Fig. S1†). The hydrodynamic diameter of UCNP@SIPO in chloroform (*D*_h_ ∼60 nm; Table 1) was smaller than that of the washed particles in water due to the reduction of the solvation layer on the former hydrophobic particles. A PD value of 0.18 indicated that the modification did not change the particle size distribution. According to TGA, the amount of SIPO on the surface of UCNPs was ∼15 wt% (Fig. 3a). In the FTIR spectrum, SIPO exhibited peaks at 1099 and ∼1150 cm^−1^ ascribed to the C–O–C ether and ester groups, peaks at ∼1600 and ∼1728 cm^−1^ assigned to the C=C and C=O groups and a peak at ∼2900 cm^−1^ belonging to the CH groups (Fig. 3b).

### Miniemulsion polymerization of GMA with UCNP@SIPO dispersion

The KPS/NaAs-initiated RAFT miniemulsion polymerization of GMA at 50 °C was selected for the encapsulation of UCNPs (Fig. S2†), offering opportunities not afforded by conventional emulsion polymerization.^36^ This involved the use of an efficient macroRAFT surfactant/CTA system synthesized from GMMA and CPCTPA (Fig. 1a) to form very small (0.08–0.30 μm) monomer droplets serving as self-contained nanoreactors. The absence of residual low-molecular-weight surfactants may contribute to future particle biocompatibility. In contrast to conventional radical reactions, RAFT allowed to control the polymerization and obtain moderate molecular weight (*M*_n_ = 11 kg mol^−1^) with a narrow distribution (*M*_w_/*M*_n_ = 1.1), which is characteristic of RAFT and essential for bioapplications.^37^ The structure of all reaction components was confirmed by ^1^H NMR spectroscopy (Fig. S3†). Next, the reaction kinetics of miniemulsion polymerization (10 wt% GMA) in the presence and absence of UCNPs was studied to determine the effect of particles. In both cases, monomer conversion increased rapidly during the first 45 min and reached 98% after 2 h (Fig. S4a†). After 15 min of induction time, the dependence of ln([*M*_0_]/[*M*]) on time was linear and followed pseudo-first-order kinetics, indicating a constant number of radicals and good control of polymerization (Fig. S4b†). This was consistent with the typical reaction kinetics of RAFT emulsion or dispersion polymerization.^38,39^ No effect of UCNPs on the polymerization rate was observed. The high molecular weight of the polymer (*M*_n_ = 5 × 10^5^ kg mol^−1^) can be explained by intramolecular crosslinking of the chains *via* hydrolysis of the oxirane groups.

**Fig. 4:**
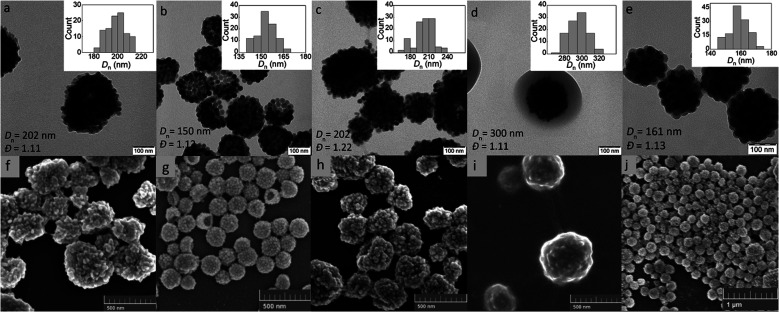
(a–e) TEM and (f–j) SEM micrographs of UCNP@PGMA (a–d and f–i) and UCNP@PGMMA particles (e and j); UCNP/GMA = 0.1 (a and f), 0.5 (b and g), 0.7 (c and h) and 1 w/w (d and i).

An important parameter affecting the properties of UCNP@PGMA particles, including their size and morphology, is the amount of hydrophobic agent added to the miniemulsion polymerization. According to TGA, the amount of SIPO on the UCNP surface reached 15 wt% (*i.e.* UCNP/SIPO = 5.7 w/w), which was not sufficient to stabilize the miniemulsion. Therefore, additional SIPO was added to the polymerization system (UCNP/SIPO was then 3 w/w) to achieve not only good colloidal stability but also control the size, morphology and reproducibility of the synthesis; in subsequent experiments, the ratio UCNP/SIPO = 3 w/w remained constant. A further important variable in the miniemulsion polymerization was the ratio of reaction components.^40^ With increasing UCNP/GMA ratio (0.1, 0.5, 0.7 and 1 w/w), the morphology of UCNP@PGMA particles analyzed by TEM and SEM changed from irregular (Fig. 4a and f) to spherical (Fig. 4b–d and g–i) and from hollow raspberry to compact raspberry morphology. A similar raspberry structure has been previously reported in the literature for iron oxide nanoparticles^41^ and for well-defined hybrid silica particles (∼250 nm) with polystyrene latex on the surface prepared by miniemulsion polymerization.^42^ In considering a possible mechanism for the formation of the raspberry-like morphology of UCNP@PGMA, we assume that the hydrophobic UCNP@SIPO particles formed PGMA-encapsulated assemblies that were stabilized by sulfate and carboxylate ions from KPS and macroRAFT agent, respectively. As for the hollow particles prepared at UCNP/GMA ratios of 0.5 and 0.7 w/w, their structure may originate from the phase separation in the miniemulsion droplets between PGMA, which tends to migrate to the oil–water interface, and hydrophobic UCNP@SIPO (Fig. 4g and h). SIPO thus acts not only as a particle coating, but also as a porogen. Such a mechanism has been described elsewhere for polymerization-induced self-assembly (PISA),^43^ redox-initiated RAFT-mediated PISA^44^ and nitroxide-mediated miniemulsion copolymerization of styrene and divinylbenzene.^45^ According to TEM, the smallest UCNP@PGMA particle size (*D*_n_ = 150 nm) with relatively low dispersity (*Đ* = 1.12) was obtained at UCNP/GMA ratio = 0.5 w/w (Fig. 4b and g); this ratio was used in further experiments. This narrow particle size distribution can be attributed to the short nucleation time in the monomer droplets, as longer droplet nucleation times are known to broaden the particle size distribution.^21^ The particle size *D*_n_ further increased with increasing UCNP concentration in the polymerization feed, which was in agreement with the DLS results. This can be explained by the inhibition of polymerization by Ln^3+^ ions, which act as radical scavengers, reducing the polymerization rate and molecular weight of the polymer, thus supporting the merging of primary particles. However, there were differences between the TEM and DLS values of UCNP@PGMA particles (*D*_n_ = 150 nm, *D*_h_ = ∼260 nm, PD = ∼0.28 and *ζ*-potential = −20 mV; Table 1), which can be explained by the presence of solvation layer around the particles and their raspberry-like morphology; the negative *ζ*-potential can be attributed to carboxyl and/or sulfate ions originating from macroRAFT agent and/or KPS, respectively. The chemical composition of PGMA coating on UCNPs was characterized by FTIR spectroscopy, which showed peaks at 1150 and 1728 cm^−1^ assigned to C–O–C ester and C=O groups in PGMA; the peak at 910 cm^−1^ belonged to its oxirane groups and the peaks at ∼2900 and 3444 cm^−1^ were attributed to CH and OH groups, respectively (Fig. 3b). According to the TGA, the amount of PGMA on the particles was ∼30 wt%, and together with SIPO, the organic coating content was ∼45 wt% (Fig. 3a).

### UCNP@PGMMA particles

**Fig. 5:**
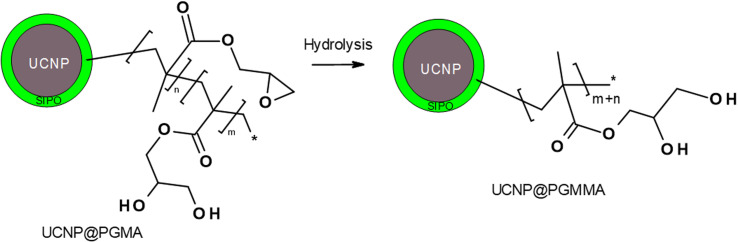
Scheme of hydrolysis of UCNP@PGMA particles.

Finally, the hydrophilic UCNP@PGMMA particles were prepared by acid-catalyzed ring-opening hydrolysis of the oxirane groups of the rather hydrophobic UCNP@PGMA particles (Fig. 5). The number-average UCNP@PGMA diameter (*D*_n_ = 150 nm) slightly increased after hydrolysis to UCNP@PGMMA (*D*_n_ = 161 nm; Fig. 4e and j), which can be explained by partial crosslinking during the oxirane ring opening. The dispersity and morphology of the particles did not change significantly, which was also confirmed by DLS measurements (Table 1). The FTIR spectrum of UCNP@PGMMA also confirmed the successful conversion of hydrophobic PGMA to hydrophilic PGMMA, as the peak corresponding to the vibration of the oxirane groups of GMA at 910 cm^−1^ disappeared; moreover, the intensity of the broad peak of the hydroxyl groups at 3444 cm^−1^ increased (Fig. 3b). The peaks at ∼2900, 1728 and ∼1150 cm^−1^ belonged to CH, C=O and C–O–C ester groups. According to TGA, the UCNP@PGMMA particles had a total ∼50 wt% polymer including both PGMMA and SIPO (Fig. 3a). The presence of UCNPs in PGMA or PGMMA particles was also confirmed by fluorescence emission spectroscopy (Fig. 3c), which showed typical emission of NaYF_4_:Yb^3+^,Er^3+^ at 547 and 660 nm after excitation with 980 nm laser.^9^ This indicates that particle surface modification did not affect the emission wavelengths. Comparing neat and encapsulated UCNPs with the same concentration of luminophore (4 mg ml^−1^), it was found that the polymer coatings (∼50 wt% according to TGA) reduced the upconversion emission intensity by approximately twice (Fig. 3c). However, the fluorescence was still high enough to visualize UCNP@PGMMA or UCNP@PGMA particles in applications.

### Chemical stability of UCNP@PGMA and UCNP@PGMMA particles

**Fig. 6:**
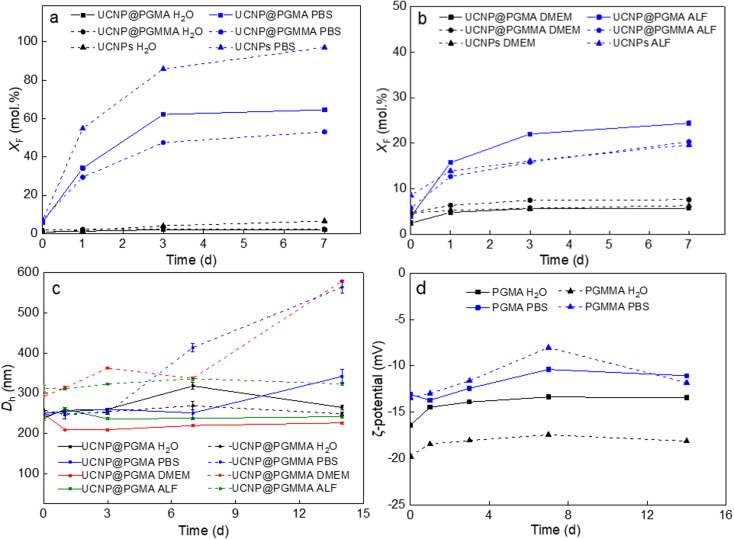
(a and b) Time dependencies of the F^−^ ion molar fraction (*X*_F_) in the supernatants after incubation of particles in different media at 37 °C, (c) the hydrodynamic diameter *D*_h_ and (d) *ζ*-potential of particles in water, PBS (pH 7.4), DMEM and ALF.

Recent studies have shown that lanthanide-based UCNPs are susceptible to dissolution in aqueous media, especially at higher temperatures due to the higher solubility of NaYF_4_, which can reduce the intensity of luminescence and induce cell death.^46–48^ The disintegration of the nanoparticles was concentration-dependent, with the fluoride ion playing a major role in the disintegration.^49^ In addition, it was found that coating UCNPs with polysulfonates not only prevented dissolution of UCNPs and preserved the upconversion emission, but also provided chemical stability in highly acidic environment.^50^ Therefore, the chemical stability of our polymer-encapsulated UCNPs in different media (water, PBS, DMEM and ALF) was evaluated at 37 °C, which simulates the temperature in *in vitro* experiments (Fig. 6). As expected, the particles dissolved least in water and most in PBS due to the accelerated hydrolysis of the lanthanide surface atoms by complexation with phosphate ions from PBS.^47^ The high resistance of both UCNP@PGMA and UCNP@PGMMA particles dispersed in water for 7 days to dissolution was confirmed by a small *X*_F_ ∼2 mol%, which was much lower than that of UCNPs (*X*_F_ = 6.5 mol%; Fig. 6a). In contrast, under the same conditions in PBS, 97, 65 and 53 mol% of F^−^ ions were released from UCNPs, UCNP@PGMA and UCNP@PGMMA, respectively (Fig. 6a). This shows that both polymer coatings visibly inhibited the particle dissolution in PBS. The 12 mol% difference in the amount of F^−^ ions released from UCNP@PGMA and UCNP@PGMMA particles can be attributed to the crosslinking of the PGMMA shell, which slows the diffusion of phosphates to the particle surface, thus retarding the dissolution. Nevertheless, compared to poly(ethylene glycol)-alendronate and poly(*N*,*N*-dimethylacrylamide-*co*-2-aminoethylacrylamide)-alendronate coatings, PGMA and PGMMA provided 22% and 36% better protection against UCNP dissolution, respectively.^51^

The chemical stability of UCNP@PGMA and UCNP@PGMMA particles under *in vitro* conditions was further simulated by aging them in DMEM and ALF, which resembles the lysosomal environment after endocytosis. After 7 days of aging of UCNPs, UCNP@PGMA and UCNP@PGMMA particles in DMEM at 37 °C, *X*_F_ was 6.3, 5.8 and 7.6 mol%, respectively, while in ALF, 19.7, 20 and 24 mol% of F^−^ ions were released in the same time, suggesting that degradation would occur predominantly inside the lysosome of the living cells (Fig. 6b).

### Dispersion stability of UCNP@PGMA and UCNP@PGMMA particles

The dispersion stability of our polymer-encapsulated UCNPs in water, PBS, DMEM and ALF at 37 °C was evaluated by measuring their hydrodynamic diameter *D*_h_ (Fig. 6c). The *ζ*-potential was determined only in water and PBS because the components of DMEM and ALF, including amino acids and vitamins, interfered with the DLS measurement. Both UCNP@PGMA and UCNP@PGMMA particles were colloidally stable, *i.e.*, did not aggregate in water and ALF for at least 14 days, when no significant changes in their *D*_h_ were observed. The *D*_h_ of UCNP@PGMA in water varied between 260 and 320 nm over this time, while that of UCNP@PGMMA reached ∼250 nm. The UCNP@PGMA particles had similar stability in DMEM, while the UCNP@PGMMA particles were stable here only for 7 days. *D*_h_ of UCNP@PGMA and UCNP@PGMMA in PBS increased after 7 and 3 days, respectively (Fig. 6c). Thus, both types of particles appeared to be less stable in PBS than in water, DMEM and ALF due to their interactions with phosphate buffer ions. Both PGMA and PGMMA coatings provided good dispersion stability of UCNPs in the tested media for at least 3 days, which is sufficient for most applications. The *ζ*-potential of both particle types in water was constant (−20 mV) during 14 days, while it slightly increased in PBS to −12 mV due to the formation of the counterion layer (Fig. 6d). Thus, the steric repulsion of the PGMMA macroRAFT agent seemed to contribute to the long-term stability of both dispersions in both media.

### Antifouling properties of UCNP@PGMA and UCNP@PGMMA particles

**Fig. 7:**
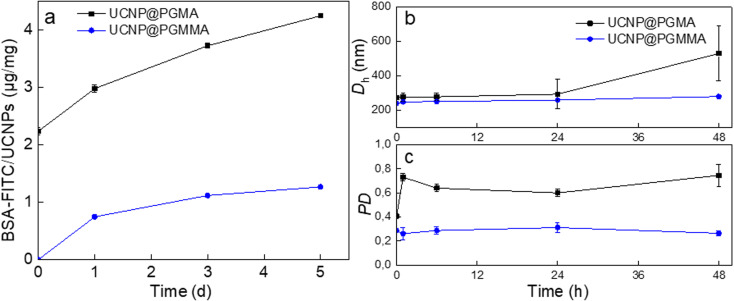
Time dependence of (a) amount of BSA-FITC adsorbed on UCNP@PGMA and UCNP@PGMMA particles and (b) hydrodynamic diameter *D*_h_ and (c) polydispersity PD of UCNP@PGMA and UCNP@PGMMA in PBS (pH 7.4; 48 h) in the presence of BSA-FITC (0.5 mg BSA per ml).

The antifouling properties of UCNP@PGMA and UCNP@PGMMA particles incubated with BSA-FITC model were evaluated by DLS and UV-vis spectrophotometry. The *D*_h_ of UCNP@PGMA slightly increased after 48 h of incubation due to BSA adsorption, whereas the *D*_h_ of UCNP@PGMMA did not change because there was no interaction with albumin. The UV-vis spectra of UCNP@PGMA particles showed a slightly increased intensity of BSA-FITC peak at 500 nm compared to the UCNP@PGMMA particles (Fig. 7a), which was consistent with the DLS analysis. The amount of BSA-FITC adsorbed on UCNP@PGMA and UCNP@PGMMA particles after 5 days was 4 and 1 μg per mg, respectively. Compared to UCNP@PGMA, UCNP@PGMMA particles thus exhibited improved antifouling performance (Fig. 7b and c). Let us note that 150 nm nanoparticles are known to circulate in the body for 5 h,^52^ so in this respect the antifouling properties of UCNP@PGMMA particles lasting at least 5 days and dispersion stability lasting for 7 days are more than sufficient. The reason for higher protein adsorption on UCNP@PGMA particles compared to UCNP@PGMMA particles can be explained by the presence of hydrophobic PGMA domains on the surface interacting with BSA.^53^ However, both types of particles showed good antifouling properties because the macroRAFT agent of the miniemulsion polymerization contained a hydrophilic PGMMA block localized on the particle surface.

### Cytotoxicity of UCNP@PGMA and UCNP@PGMMA particles

**Fig. 8:**
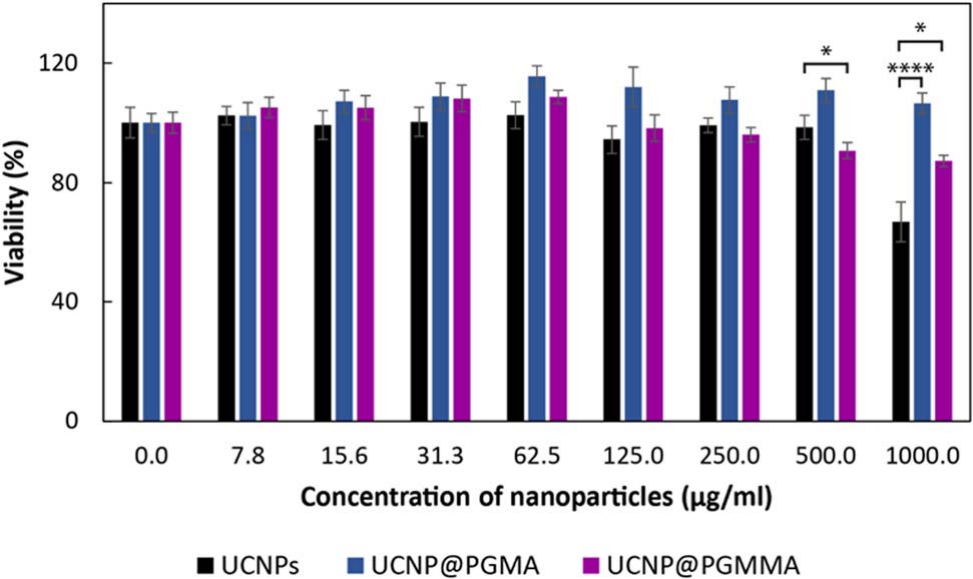
Cytotoxicity of UCNPs, UCNP@PGMA and UCNP@PGMMA particles after 24 h of incubation with human dermal fibroblasts according to MTT cell viability assay. Data represent the mean ± S.E.M. of three experiments performed in triplicate. *p < 0.05 and ****p < 0.0001 were considered statistically significant; one-way anova followed by Dunnett's post hoc test.

The advantageous physicochemical properties of UCNPs in various bioapplications are often limited by the so-called “dark” toxicity induced by the release of lanthanide and fluoride ions.^5,12^ To assess whether the polymeric coatings of UCNPs can reduce their cytotoxicity, the viability of HF cells as a model for human healthy (non-tumor) cells was tested in the presence of particles for 24 h using the MTT assay (Fig. 8). It was found that the cytotoxicity of neat UCNPs was concentration-dependent and at the highest level used (1 mg ml^−1^) the cell viability was 67%, which represents moderate cytotoxicity according to ISO 10993-5.^54^ Both PGMA and PGMMA coatings reduced the cytotoxicity of UCNPs at this concentration by ∼35% (*p* < 0.0001) and 20% (*p* < 0.05), respectively. Thus, PGMMA was slightly more toxic (87% cell viability) than PGMA (100% viability; Fig. 8). However, the main advantage of the PGMMA coating consists in its increased hydrophilicity and antifouling properties.^55^ Indeed, the protection from the adsorption of blood proteins and other blood molecules on the nanoparticle surface (antifouling) prevents opsonization, which otherwise triggers an immune response in the organism and leads to the clearance of the nanoparticles from the bloodstream. PGMMA coating enhanced the antifouling properties of UCNP@PGMA and UCNP@PGMMA particles, which may prevent protein adhesion and subsequent opsonization in a comparable way to that previously demonstrated for PEG-modified nanoparticles.^56^ This could prolong the circulation of particles in the bloodstream, making the UCNP@PGMA and UCNP@PGMMA particles promising for various biomedical applications that require low toxicity and high antifouling, such as *in vivo* deep tissue imaging or photodynamic therapy. Detailed investigation of the *in vivo* pharmacokinetics of the particles is now in progress.

## Conclusions

The advantage of UCNPs is their unique ability to convert NIR photons, which can penetrate biological tissues, into visible light emission. The excellent optical properties of UCNPs are associated with sharp emission peaks, absence of background noise, low autofluorescence, high stability and tunable excitation. However, UCNPs are currently not yet used in medical applications due to their poor chemical stability in aqueous media. Although the coating of inorganic nanoparticles with various polymers has been the subject of a number of works, the use of miniemulsion polymerization of GMA to effectively encapsulate UCNPs inside the polymer particles has not yet been described. Because PGMA containing oxirane groups is a reactive polymer, it allows various modifications by nucleophilic ring opening to form a variety of specific derivatives. Oxirane groups can be easily transformed to amines, aldehydes, sulfonates, chelates and other functional groups to bind diagnostic or therapeutic agents.^57^

In this work, we have investigated the RAFT miniemulsion polymerization of GMA initiated by KPS/NaAs using a macroRAFT chain transfer agent, which also served as a surfactant to stabilize the miniemulsion. The polymerization was carried out in the presence of SIPO hydrophobe and SIPO-modified UCNPs. By adjusting the content of UCNP@SIPO in the monomer droplets, it was possible to control the morphology of PGMA particles. Under elevated UCNP content (UCNP/GMA = 1 w/w), a well-packed core–shell morphology was prepared, whereas under low UCNP content (UCNP/GMA ≤ 0.7 w/w), a large number of holes was formed on the surface of the raspberry-like particles. The hollow structure, which can significantly reduce mass transfer resistance and has a high drug loading capacity for controlled release, was supposedly formed by phase separation and polymerization-induced self-assembly due to the incompatibility of the growing PGMA chains and SIPO-stabilized UCNPs. The relatively low polymerization temperature allowed for improved nucleation of the monomer droplets, which had a positive effect on the stability of the miniemulsion.^42^ In addition, the resulting UCNP@PGMA particles had a relatively narrow size distribution, ensuring the same physical, chemical and biological properties and reproducibility of results in applications.

Subsequently, the reactivity of PGMA was exploited for the acid-catalyzed hydrolysis of UCNP@PGMA to UCNP@PGMMA particles. Both PGMA and PGMMA coatings significantly improved the chemical and dispersion stability of the UCNPs both in water and biological media while providing antibiofouling properties and no toxicity. In particular, the hydrophilic PGMMA coating provided better antifouling properties compared to PGMA. This low biofouling and superb cytocompatibility, combined with relatively small particle size and distribution, together with their excellent luminescence properties, high chemical and dispersion stability, make these particles promising candidate for precision theranostic bioapplications such as drug delivery systems, cell and tissue imaging, biosensing, deep *in vivo* tissue imaging, and photodynamic tumor therapy.

## Author contributions

TV – investigation, VP – formal analysis, MF – methodology, RLK – validation, DH – supervision and writing.

## Conflicts of interest

There are no conflicts to declare.

## Supplementary Material

NA-005-D3NA00793F-s001
