# Supplementary material for: Poly(glycerol monomethacrylate)-encapsulated upconverting nanoparticles prepared by miniemulsion polymerization: morphology, chemical stability, antifouling properties and toxicity evaluation
Source: Nanoscale Adv. 2023 Nov 13;5(24):6979–89. doi: 10.1039/d3na00793f (PMC10697003; doi:10.1039/d3na00793f)
Supplement: NA-005-D3NA00793F-s001 [file NA-005-D3NA00793F-s001.pdf]

Electronic Supplementary Material (ESI) for Nanoscale Advances.
This journal is © The Royal Society of Chemistry 2023

Electronic supplementary information

Poly(glycerol monomethacrylate)-encapsulated upconverting nanoparticles prepared by miniemulsion polymerization: Morphology, chemical stability, antifouling properties and toxicity evaluation

Taras Vasylyshyn, Vitalii Patsula, Marcela Filipová, Rafal Lukasz Konefal, Daniel Horák*

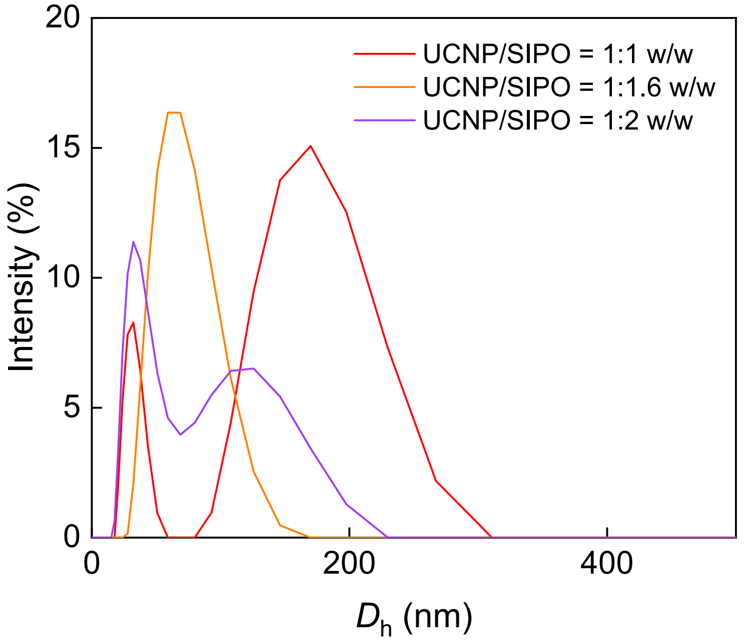

Figure S1. UCNP@SIPO particle size distribution in chloroform at UCNP/SIPO ratio (a) 1:1, (b) 1:1.6 and (c) 1:2 w/w according to DLS.

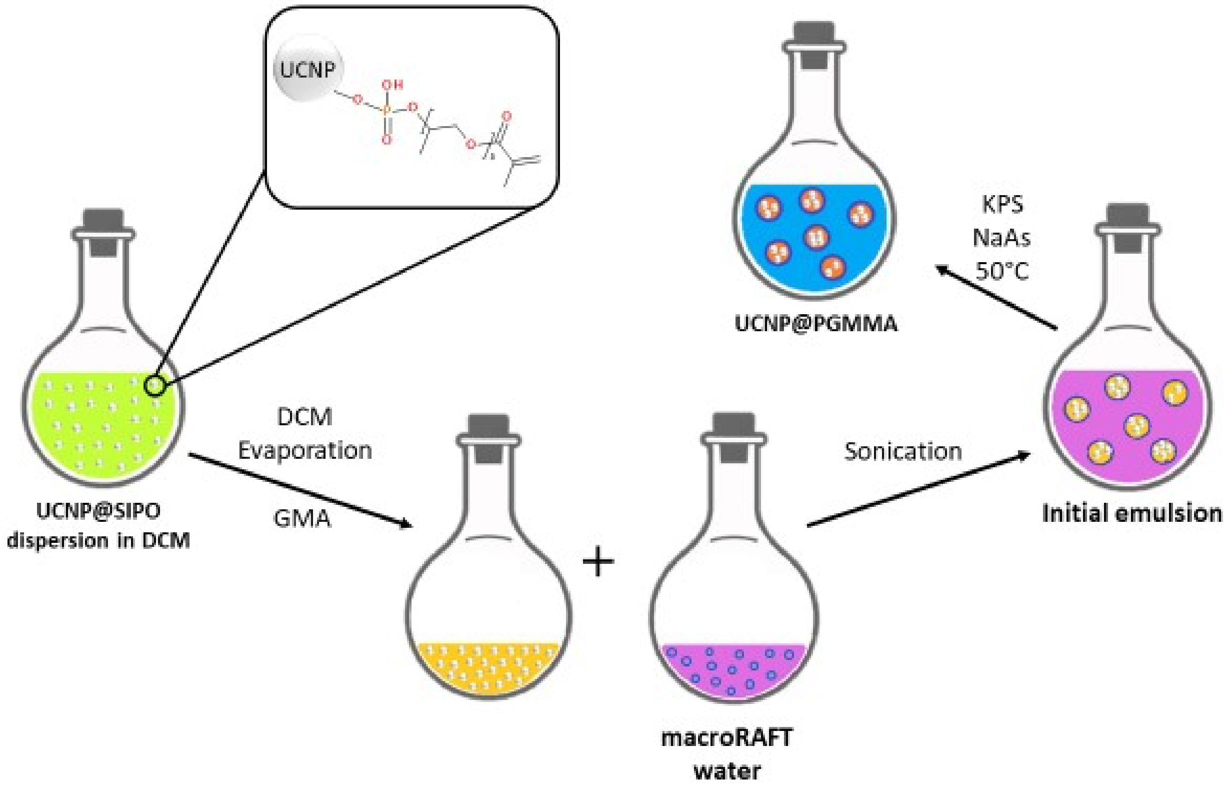

Figure S2. RAFT miniemulsion polymerization of GMA in the presence of UCNPs.

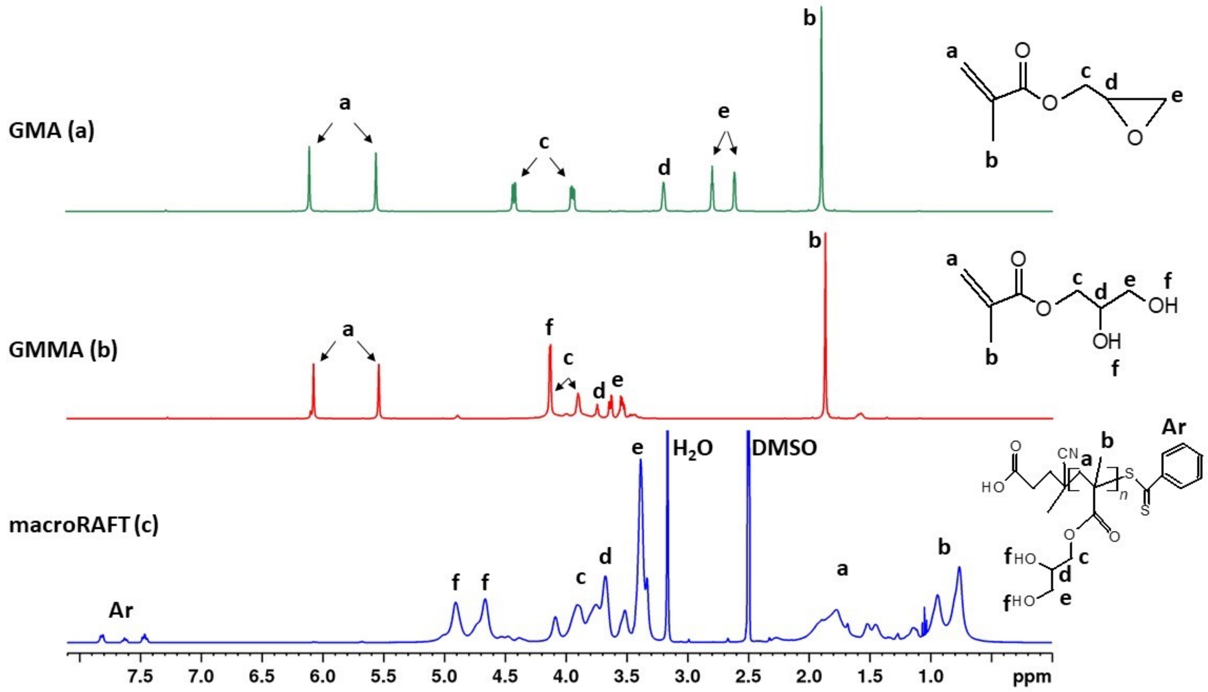

Figure S3. ^{1}H NMR spectra of (a) GMA, (b) GMMA and (c) macroRAFT agent.

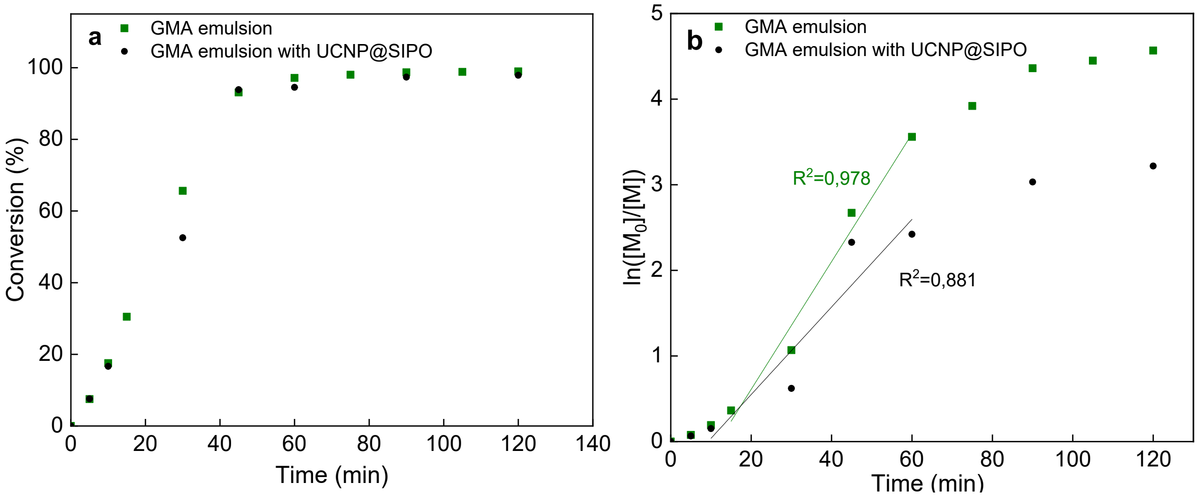

Figure S4. Time dependence of (a) GMA conversion and (b) ln([M_0]/[M]) to demonstrate kinetics of RAFT emulsion polymerization of GMA in the absence or presence of UCNP@SIPO particles.
